# Exploring Bioactive Compounds from Fruit and Vegetable By-Products with Potential for Food and Nutraceutical Applications

**DOI:** 10.3390/foods14223884

**Published:** 2025-11-13

**Authors:** Filomena Carvalho, Radhia Aitfella Lahlou, Luís R. Silva

**Affiliations:** 1 Sport Physical Activity and Health Research e Innovation Center (SPRINT), Polytechnic of Guarda, 6300-559 Guarda, Portugal; filomenacarvalho@ipg.pt (F.C.); radhialahlou@ipg.pt (R.A.L.); 2 RISE-Health, Faculty of Health Sciences, University of Beira Interior, 6201-506 Covilhã, Portugal; 3 Chemical Engineering and Renewable Resources for Sustainability (CERES), Department of Chemical Engineering, University of Coimbra, 3030-790 Coimbra, Portugal

**Keywords:** fruit and vegetable by-products, bioactive compounds, green extraction techniques, nutraceuticals

## Abstract

The increasing production of fruit and vegetable by-products from the food processing industry presents both environmental challenges and opportunities for valorisation as sources of bioactive compounds. These by-products, including peels, seeds, pomace, and leaves, are rich in polyphenols, carotenoids, dietary fibres, glucosinolates, phytosterols, and essential oils, which exhibit antioxidant, anti-inflammatory, antimicrobial, and prebiotic activities. Recent advances in green extraction technologies, including ultrasound-, microwave-, supercritical fluid-, and cold plasma-assisted extraction, allow for an efficient and sustainable recovery of these compounds, while preserving their bioactivity. Incorporation of by-product-derived extracts into functional foods and nutraceuticals offers health-promoting benefits and supports circular bioeconomy strategies. However, challenges remain in standardisation, safety assessment, and regulatory approval, among others. This review summarises current progress and outlines future directions for the sustainable utilisation of fruit and vegetable by-products in health-oriented applications.

## 1. Introduction

Over the past decade, the food processing industry has grown significantly, establishing itself as one of the fastest-growing sectors globally [1]. However, food waste is a major global challenge that results from food processing. Approximately one-third of all food produced, around 1.6 billion tons, is lost or wasted, resulting in approximately 3.3 billion tons of CO_2_ emissions, according to estimates by Baysal and Ülkü [2] and FAO [3]. Fruit and vegetable processing, especially in the juice industry, generates substantial amounts of waste, like leaves, peels, seeds, excess pulp, damaged or rejected fruits, and stones. While often discarded, these by-products are rich in valuable bioactive compounds [4]. Edible plants are rich in a diverse selection of naturally occurring compounds, with over 5000 identified so far, including groups such as alkaloids, carotenoids, polyphenols, and sulfur- and nitrogen-based substances. These bioactive compounds play a significant role in health, and research consistently links diets rich in them to a lower chance of developing chronic illnesses such as heart disease, various types of cancer, type 2 diabetes, Alzheimer’s, joint degeneration, and even vision problems like macular degeneration [5,6,7].

Despite their proven health benefits, these valuable compounds are often lost when fruit and vegetable by-products are discarded. Recognizing the potential of these residues, researchers and industry stakeholders have increasingly focused on strategies to recover and utilize these bioactive compounds [8,9,10]. To utilize this potential, the integration of advanced green technologies into sustainable waste valorisation processes is essential [11]. Eco-friendly extraction methods, such as ultrasound-assisted extraction (UAE), microwave-assisted extraction (MAE), and supercritical fluid extraction (SFE), are emerging as sustainable alternatives to traditional techniques. These innovative approaches are designed to reduce environmental impact while still efficiently extracting valuable compounds [12]. For example, UAE utilises ultrasonic waves to enhance the release of valuable compounds into the extraction solvent, requiring low energy input, short processing times, and low temperatures, thereby reducing environmental impact and making it a greener choice compared to conventional methods [13].

The health-promoting effects of these extracted compounds make them attractive for use in functional foods and dietary supplements. Simultaneously, consumers’ awareness of the link between diet and health has caused a global shift toward functional food consumption [14]. For example, bitter orange or lemon peels have been used to fortify kefir, resulting in an increased antioxidant activity of the product [15]. Grape pomace has been incorporated into various functional foods, including baked goods, dairy products, smoothies, and snack bars, to enhance their nutritional value and provide health benefits [16]. Tomato skin and seeds can be used to improve the nutritional value and provide natural antioxidants in meat products [17].

Therefore, the valorisation of fruit and vegetable by-products through advanced extraction technologies not only contributes to waste reduction and environmental sustainability but also supports the development of innovative functional foods. This approach aligns with the principles of a circular bioeconomy and meets the growing demand from consumers for natural, health-promoting ingredients [18].

Unlike recent reviews that focus either on the chemical composition of by-products or on specific extraction methods, this review integrates both compound diversity and technological perspectives, comparing how different green extraction approaches influence the recovery of bioactive compounds and highlighting their practical translation into food and nutraceutical products. Accordingly, it provides a comprehensive overview of recent advances in the extraction, characterization, and application of bioactive compounds from fruit and vegetable by-products. Regarding the literature search, research was conducted between January 2020 and September 2025, using the Web of Science and PubMed databases, with the keywords “fruit by-products”, “vegetable by-products”, “dietary supplements”, and “bioactive compounds” combined. Studies focused on non-plant materials, or classified as conferences, abstracts, or non-peer-reviewed papers, were excluded. Review papers were also not considered, being consulted only to provide context, rather than as primary data sources.

## 2. Bioactive Compounds in Fruit and Vegetable By-Products

Fruit and vegetable by-products, including peels, seeds, pomace, stems, and leaves, are increasingly recognized as concentrated sources of bioactive compounds. These secondary metabolites, produced by plants for defence and adaptation, reveal a wide range of biological activities beneficial to human health, such as antioxidant, anti-inflammatory, antimicrobial, and anticancer effects [10,19]. Recent research has confirmed the richness of these by-products in polyphenols, carotenoids, dietary fibres, phytosterols, glucosinolates, and essential oils. Table 1 shows some examples.

The most studied group of these compounds is polyphenols. These are naturally occurring organic compounds found predominantly in plant-based foods, characterized by the presence of multiple phenol units and classified into several categories, including flavonoids, phenolic acids, tannins, and stilbenes [20,21,22]. Pomegranate peel, an abundant by-product of juice production, contains exceptionally high levels of punicalagins, an ellagitannin, with the highest yield obtained by Li et al. using ultrasonic-assisted ethanol extraction, being 505.89 mg/g dry weight [23]. Ellagic acid and gallic acid are also commonly found in this by-product, at concentrations of 50.32 to 134.36 µg/mL and 80.6 to 170.24 µg/mL, respectively [23,24]. Apple pomace contains chlorogenic acid, caffeic acid, syringin, procyanidin B2, cinnamic acid, phlorizin, hyperin and quercetin, with the main compound being procyanidin B2, at a maximum of 92.62 mg per 1 kg of dry industrial apple pomace [25].

Several fruit and vegetable by-products are rich in carotenoids, a group of tetraterpenoid compounds present in plants, algae, fungi, and some bacteria, which are responsible for the yellow, orange, and red colours in many fruits, vegetables, and flowers, and play vital roles in various biological processes [26,27,28,29]. Pumpkin peel flour contains significant amounts of carotenoids, ranging from 216.9 to 306.8 µg/g, depending on particle size and extraction methods [30]. The most important carotenoids identified in pumpkins include *β*-carotene, α-carotene, lutein, and zeaxanthin, with *β*-carotene being the predominant one [31]. Papaya pulp and peel are also rich in carotenoids, with lycopene being the most present one. Ultrasound-assisted extraction with soybean oil achieved the highest carotenoid content of up to 58.7 µg carotenoids/g oil from papaya pulp [32].

Dietary fibre is another major component of many fruit and vegetable by-products, classified into soluble (pectin, for example) and insoluble fibres (cellulose, hemicellulose, lignin) [33]. Soluble fibres aid in lowering the blood cholesterol and glucose absorption, while insoluble fibres help in intestinal regulation [34]. Banana peels contain a significant amount of total dietary fibre. A study reported that banana peel flour from the Indonesian Kepok variety contains 47.08 g/100 g of total dietary fibre [35]. Orange waste, primarily orange peel waste derived from the juice extraction process, is a significant source of dietary fibre. Flours derived from orange juice by-products contain between 42.44% and 62.74% total dietary fibre, with soluble and insoluble fibres varying from 5.04 to 19.95 and 23.96 to 57.70%, respectively [36]. Another study found that powdered orange residues have up to 64% total dietary fibre [37].

The lipid fraction of some by-products is also of interest, particularly for its phytosterol content, which includes plant-derived sterols that can lower cholesterol levels [38,39]. Studies have shown that oils extracted from orange seeds contain substantial amounts of phytosterols (1304.2 mg/kg) along with other bioactive compounds, such as carotenoids and tocopherols [40]. Tomato seed oil is also rich in phytosterols, including sitosterol, cycloartanol, and stigmasterol, with the supercritical CO_2_ extraction method yielding the highest phytosterol content [41].

Cruciferous vegetable by-products are particularly valuable for their glucosinolate content, a sulfur-containing secondary plant metabolite known for its anticancer and cardiovascular activities [42]. Industrial broccoli by-products are rich in glucosinolates, with glucoraphanin being the primary type, accounting for 32–64% of the total glucosinolates, depending on the cultivar [43]. Cauliflower by-products, including leaves and stalks, also contain glucosinolates, which are variable depending on the part of the plant [44].

Lastly, essential oils, complex mixtures of volatile compounds, primarily terpenes (mono-, sesqui, and diterpenes) and phenylpropanoids, are highly valued for their aromatic and therapeutic properties [45,46,47]. D-limonene, α-pinene and linalool and common essential oil components found in fruit and vegetables by-products [48,49,50]. Citrus fruits’ by-products, particularly their peels, are a rich source of essential oils. For example, the peel of *Citrus grandis* Osbeck cv. Mato Peiyu contains essential oils with a content ranging from 0.76% to 1.34%, primarily composed of monoterpenes like limonene and *β*-myrcene [51]. Similarly, grapefruit peel essential oil is rich in monoterpene hydrocarbons, with limonene being the major component [52].

Collectively, these studies highlight the notable diversity and concentration of bioactive compounds in fruit and vegetable by-products. The valorisation of these materials not only addresses environmental concerns associated with food waste but also provides a sustainable source of health-promoting ingredients for the food and nutraceutical industries.

The concentration and composition of bioactive compounds in by-products are influenced by several factors, including plant variety, maturity stage, agricultural practices, and processing conditions [53]. Similarly, drying and storage conditions can affect the stability and bioavailability of sensitive compounds [54]. This variability underscores the importance of standardized extraction, characterization, and quality control methods for by-product-derived ingredients.

**Table 1:** Examples of compounds present in by-products and its sources.

Class of Compounds	Source Examples	Representative Compounds	Concentrations	Ref.
Polyphenols	Pomegranate peel	Punicalagins, ellagic acid, gallic acid	50.2–134.36 µg/mL	[24]
	Apple pomace	Procyanidins B2, quercetin, hyperin	1.50–92.62 mg/kg DW	[25]
Carotenoids	Pumpkin peel	n.s.	216.9–306.8 µg/g	[30]
	Papaya pulp/peel	*β*-Carotene, lycopene, lutein	39.3–58.7 µg/g soybean oil	[32]
Dietary fibre	Banana peel	n.s.	61.8–470.8 mg/g	[35]
	Orange peel residues	Soluble fiber	5.04–19.95%	[36]
		Insoluble fiber	23.96–57.70%	
Phytosterols	Orange seed oil	n.s.	1.304 mg/g	[40]
	Tomato seed oil	Sitosterol, cycloartenol, stigmasterol	1.13–4.00 mg/g	[41]
Glucosinolates	Broccoli industrial by-products	Glucoiberin, glucoerucin, glucoraphanin	68–23,226 g/kg DW	[43]
	Cauliflower industrial by-products	Glucoiberin, glucobrassicin, sinigrin, glucoraphanin	n.s.	[44]
Essential oils	Citrus and grapefruit peel	Monoterpenes, sesquiterpenes	1.72–96.96%	[51]

n.s. not specified.

## 3. Extraction Technologies for Bioactive Compounds

Recovering high-value phytochemicals from fruit and vegetables requires selecting an efficient extraction method. Key parameters, such as the choice of solvent, temperature, and time, affect every extraction approach. Phenolic compounds are soluble in water due to their weak acidity and the presence of hydroxyl groups, which can form hydrogen bonds with water molecules [55]. Since they are polar protic solvents, ethanol and methanol also show high solubility for phenolic compounds [56,57]. Phenolics generally remain stable up to 100 °C. Significant degradation occurs at temperatures above 125 °C, particularly for compounds such as epicatechin, resveratrol, and myricetin, which are influenced by the chemical structure of these compounds [58]. Traditionally, solvent-based methods, such as maceration or Soxhlet extraction, have been used [59]. In Soxhlet extraction, dried plant material is repeatedly washed with a solvent in a Soxhlet apparatus. This exhaustive approach ensures complete extraction but typically uses large amounts of solvent and high temperatures, which may degrade some compounds [59,60,61]. Maceration is a cost-effective and straightforward method in which plant material is soaked in a solvent, such as ethanol or methanol, for an extended period. It is performed at room temperature, which makes it suitable for heat-sensitive compounds; however, it is time-consuming and also requires large quantities of solvent [59,60]. These conventional methods can degrade heat-sensitive bioactives. In phenolic extraction, Soxhlet and maceration extractions resulted in yields of only 9.68% and 8.12%, respectively, in *Annona muricata* seeds, which is significantly lower than the yields obtained with novel methods used in the same study [62].

Novel or “green” extraction techniques have been developed to reduce processing, energy use and lower solvent usage, consistent with sustainable development strategies [63]. These methods include MAE, UAE, supercritical fluid extraction, pressurised liquid extraction, and cold plasma-assisted extraction, among others [63]. The main mechanism behind UAE is acoustic cavitation. It involves the creation, expansion, and implosion of microscopic bubbles within the extraction medium. When these bubbles implode, they produce intense localized heat, strong shear forces, and micro-jets of liquid, which work together to break down cell walls and significantly improve mass transfer during the extraction process [64]. This method significantly reduces extraction time and solvent usage, while increasing yield. For example, the extraction time for Ginkgo biloba flavonol glycosides was reduced from 11.8 h to 63.6 min by using UAE [65]. UAE applied to date press cake (*Phoenix dactylifera* waste) at 40 °C with 60% ethanol for 15 min yielded 121.7 mg GAE/g total phenolics and 446.7 mg QE/g flavonoids, with great antioxidant activity [66]. Similarly, it is possible to extract up to 95% of polyphenols from floral sources under optimized conditions, using this method [13]. The mild operating temperatures, generally less than 50 °C, help preserve sensitive bioactives that would degrade using heat-based methods [67]. UAE is also very versatile, and it can be applied to a wide range of materials, including plants, algae, food by-products and environmental samples [67,68,69]. Challenges that remain are the substantial investment in ultrasonic equipment and the cost of scaling up UAE for industrial applications [70].

MAE is a modern and efficient technique that directly heats the sample by causing water molecules within the plant material to vibrate, resulting in rapid heating and cell rupture. It enhances the solubility and diffusion of target compounds into the solvent [71]. This increase in the kinetics of extraction leads to faster extraction rates and higher extraction efficiency [71]. Compared to traditional methods, MAE results in higher extraction yields, lower solvent consumption, and shorter extraction times, making it a cost-effective and environmentally friendly option [71,72]. This method avoids the degradation of compounds, resulting in higher polyphenol content and antioxidant capacity compared to conventional extraction methods performed at the same temperature profile [73]. A study compared MAE with maceration, Soxhlet and UAE, and the research concluded that MAE had the highest total phenolics and total flavonoid content extracted from *Oroxylum indicum* leaves, with 45.67 mg GAE/g and 76.82 mg QE/g, respectively, a 70% higher yield [74]. While MAE is effective at the laboratory scale, scaling up the process for industrial applications presents challenges such as, maintaining uniform heating and ensuring regulatory compliance [75,76].

Supercritical fluid extraction is a separation technique that operates above the critical temperature and pressure of a solvent, most commonly CO_2_, where it exhibits both the diffusion capacity of a gas and the dissolving power of a liquid. This unique combination allows the supercritical fluid to penetrate solid plant matrices effectively, while dissolving target compounds with high efficiency [77,78]. CO_2_ is especially attractive due to its non-toxicity, recyclability, and mild critical conditions (T = 31.2 °C, P = 72.9 atm) [77], making this method a green alternative, as it avoids the use of harmful organic solvents and leaves no solvent residues in the final product [79]. This technique also enables the selective extraction of both polar and non-polar compounds by precise control over conditions such as pressure, temperature, and co-solvents, resulting in high purity and yield of the desired extracts [80,81]. It is widely used for extracting bioactive substances from plant residues, including pomace, seeds, skins, and other agricultural byproducts [82]. The best conditions for extracting bioactive compounds from pineapple residue were found to be 20 MPa, 60 °C, and 15% ethanol as a co-solvent [83]. Similarly, the extraction of carotenoids from various vegetable matrices was optimized at 59 °C and 350 bar, with 15.5% ethanol as a co-solvent [84].

Pressurised liquid extraction is another technique that uses solvent extraction at high temperatures and pressures, consistently below the critical points, to maintain the solvent in a liquid state during the extraction procedure. Using specific pressure and temperature conditions alters the physicochemical properties of the extraction solvent, enabling easier and deeper penetration into the matrix being extracted [85]. The method is considered environmentally friendly, as it generates small volumes of waste and reduces costs and time. However, it involves the use of elevated temperatures and pressures, which may not be suitable for thermally sensitive compounds [86]. A pressurised liquid, used under optimal conditions of 75% ethanol for 11 min at 20 °C, has been employed to recover phenolic compounds from grape seed by-products, yielding a high phenolic content and antioxidant activity [87].

The last example mentioned is cold plasma-assisted extraction. This technology utilises cold plasma, a partially ionised gas that contains energy and reactive species, including reactive oxygen and nitrogen species, ozone, ions, free radicals, and ultraviolet radiation [88]. Treatment with cold plasma can cause cell disruption due to the generation of reactive species and enhance solvent penetration, thereby improving the extraction yield [89]. Cold plasma can be combined with other extraction methods, such as ultrasound-assisted extraction, to enhance the recovery of bioactive compounds further. For instance, a study on Cornelian cherry pomace demonstrated that cold plasma pretreatment improved the extraction efficiency when combined with ultrasound [90].

**Figure 1:**
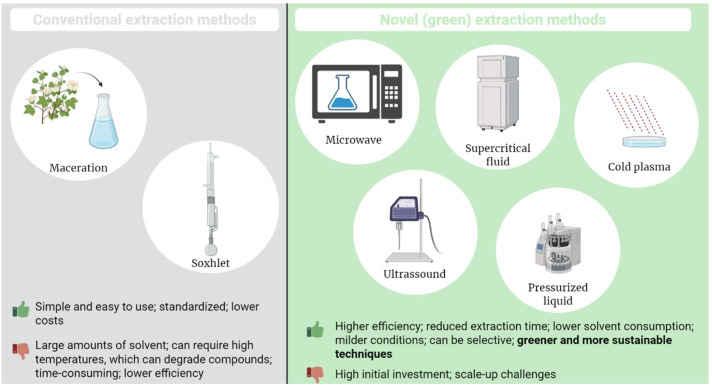
Conventional and novel methods of bioactive compounds extraction.

While conventional techniques remain widely used due to their simplicity, they are often limited by low efficiency and high solvent consumption. In contrast, modern extraction methods offer more sustainable, efficient, and targeted approaches, aligning with current demands for greener processes (Figure 1).

Recent evidence suggests that UAE, MAE, and SFE present distinct trade-offs in terms of yield, energy/speed, and sustainability. UAE enhances mass transfer via acoustic cavitation and can deliver high phenolic yields under mild conditions. For instance, pomegranate peel punicalagin reached 505.89 mg/g DW in 25 min with ethanolic UAE, while preserving bioactivity [23]. MAE frequently outperforms conventional techniques: in *Oroxylum indicum* leaves, this technique achieved 45.67 mg GAE/g (TPC) and 76.82 mg QE/g (TFC), which is higher than the Soxhlet method, in significantly shorter times [74]. Direct comparisons indicate the ranking can be matrix-dependent. For apple (“Bravo de Esmolfe”), MAE matched the best conventional extract and outperformed UAE for TPC, underscoring that solvent–matrix interactions and heating profiles are key parameters in the performance [91]. SFE with supercritical CO_2_ offers solvent-free extracts and high selectivity, as demonstrated by optimised carotenoid recovery at 59 °C and 350 bar using 15.5% ethanol as a co-solvent [84]. A recent life-cycle assessment compared three extraction routes for flavonoids from *Ginkgo biloba* leaves: heat reflux extraction (HRE), UAE, and extraction using deep eutectic solvents (DES). Among the eight midpoint indicators evaluated under the ReCiPe 2016 methodology, UAE with ethanol achieved the lowest overall environmental impact, with values 10–80% lower than those of HRE and DES for all categories except water consumption. It was mainly due to UAE’s higher extraction yield, shorter processing time, and lower solvent requirement. DES-based extraction had the lowest water use, but the highest freshwater ecotoxicity and human carcinogenic toxicity. The HRE method caused the greatest impacts in most indicators. In every scenario, solvent production was the dominant contributor to the total environmental footprint. The study also found that using ethanol derived from sugarcane substantially reduced environmental impacts compared with maize-based ethanol, showing that the solvent’s origin strongly affects sustainability outcomes. Overall, the work demonstrated that conventional UAE using bioethanol currently represents the most eco-efficient option [92].

## 4. By-Products of Fruit and Vegetables in Food and Nutraceutical Applications

The valorisation of fruit and vegetable by-products as sources of bioactive compounds has emerged as a promising strategy in the development of nutraceuticals and dietary supplements. Building upon the compositional data in Section 2, this section summarises how those bioactive-rich extracts can be applied in food and nutraceutical products (Table 2).

Extracted through UAE, polyphenols have been shown to exhibit strong antioxidant activity, supporting their inclusion as functional ingredients in dietary supplements [93,94]. By-products such as cantaloupe residues are enriched with polyphenols and dietary fibre and were incorporated into gluten-free doughnuts. Their antioxidant potential makes them valuable for functional food formulations [94].

*Asparagus officinalis* stems and roots contain dietary fibres, fructans, and polyphenols that exhibit promising prebiotic effects, particularly in modulating gut microbiota, due to their ability to promote the growth of probiotic bacteria while not stimulating pathogenic strains. This effect was attributed to their dietary fiber composition [95]. Turmeric starch production residues were investigated for their use as an ingredient in cookies. The turmeric by-product powder was rich in phenolics, flavonoids, curcumin, dietary fibres, and essential minerals and significantly improved the antioxidant properties of standard cookies [96]. Similarly, oat by-products, husk and bran, were tested as raw materials for the development of fibre-rich preparations with antioxidant properties. Oak husk contained a higher total phenolic acid concentration, with ferulic acid as the main compound. A mixture with 60–70% husk and 30–40% bran provided 60–70% of fibre and significantly increased oxidant activity [97]. Grape pomace, bilberries, and red currants are well-studied for their high content of phenolics, including chlorogenic acid, rutin, catechin, and ferulic acid. Their transformation into powdered and encapsulated supplements highlights their potential in preventing oxidative stress-related chronic diseases [98]. In another example, apple by-products subjected to lactic acid fermentation have demonstrated not only antioxidant capacity but also potential hypoglycaemic and antidiabetic effects, broadening their nutraceutical relevance [99].

Vegetable by-products such as artichoke are also rich in hydroxycinnamates and flavonoids (Table 2). Aqueous extraction of these compounds highlights their dual role as antioxidants and potential prebiotics, with relevance in both nutraceutical and dietary supplement applications [100]. *Mauritia flexuosa* extracts have shown notable anti-inflammatory effects, underlining the therapeutic potential of tropical fruit residues [101]. Citrus peels and pomace, particularly from *Citrus reticulata* Blanco, are recognised for their catechins, neohesperidin, and nomilinic acid derivatives, which contribute strong antioxidant properties. Extracted through UAE, these compounds can be applied as functional ingredients in both nutraceutical and food products [102]. Wongkaew et al. explored the valorisation of mango peel as a source of bioactive compounds with prebiotic potential. The study employed enzymatic hydrolysis using pectinase to extract pectic oligosaccharides under optimized conditions (0.3% pectinase, 24 h), resulting in oligosaccharides with an average molecular weight of 643 Da (Table 2). These compounds exhibited significant prebiotic activity by selectively promoting the growth of beneficial gut bacteria, including *Bifidobacterium animalis* and *Lactobacillus reuteri*, highlighting the potential of mango peel as a sustainable ingredient for functional foods and dietary supplements that support gut health and overall well-being [103]. Gharibi et al. investigated pistachio hulls, a by-product of nut processing, as a source of polyphenolic compounds with potential health benefits (Table 2). They identified key compounds, including cyanidin-3-O-galactoside, gallic acid, and catechin. These extracts exhibited strong antioxidant activity, significant anti-*Candida* effects, and antiglycative properties [104]. Similarly, the phenolic compounds in hazelnut (*Corylus avellana*) shells compounds demonstrated significant antioxidant activity, as well as inhibitory effects against key enzymes involved in type 2 diabetes and hypertension [105]. These findings suggest that these nut processing by-products could be a valuable, sustainable source of bioactive ingredients for the development of functional foods and nutraceuticals aimed at managing metabolic disorders.

Guava purée by-products were evaluated as prebiotic ingredients for yoghurt. Enzymatic treatment with cellulase and xylanase enhanced hydrolysis, yielding extracts rich in rhamnose and xylose with higher prebiotic activity than untreated samples (Table 2). Incorporation into yoghurt increased lactic acid bacteria counts by up to 77.6% and improved textural properties, highlighting their potential in functional foods supporting gut health [106]. A study investigating the potential of European cranberry bush (*Viburnum opulus*) and sea buckthorn (*Hippophae rhamnoides*) berry pomace as sources of bioactive lipophilic compounds, recovered using supercritical CO_2_ extraction, identified a range of valuable constituents, including triacylglycerols, tocopherols, phytosterols, and fatty acids such as linoleic, oleic, and palmitic acids (Table 2). The extracts exhibited significant oxidative stability, enhancing the shelf life of mayonnaise formulations [107].

High-intensity ultrasound (HIUS) was used to extract proteins from pumpkin leaves, aiming to enhance the recovery of bioactive compounds from this agricultural by-product. The study found that HIUS significantly increased protein yield by optimising sonication amplitude and duration. The extracted proteins exhibited strong antioxidant and metal-chelating activities. These findings suggest that pumpkin leaf proteins, extracted via HIUS, could contribute to sustainable nutrition solutions [108]. Red pomegranate seeds, a by-product of oil extraction, were analysed as a source of bioactive peptides. By applying enzymatic hydrolysis with Alcalase, pancreatin, trypsin, and pepsin, they produced hydrolysates exhibiting significant antioxidant, antihypertensive, antidiabetic, and antibacterial activities (Table 2). The Alcalase hydrolysate (H-Al) demonstrated the highest levels of angiotensin I-converting enzyme (ACE) and dipeptidyl peptidase-4 (DPP-IV) inhibition, along with potent antibacterial effects against *Escherichia coli* and *Staphylococcus aureus* [109]. Lastly, two studies by Alañón et al. analysed mango (*Mangifera indica* L.) by-products, focusing on peel and seed kernels from three cultivars at different maturation stages. The first study on mango peels, conducted across three stages (green, ripe, and overripe), reported a total phenolic content up to 27 times higher than that of the edible pulp, identifying a diverse array of compounds, including mono- and digalloyl derivatives, gallotannins, phenolic acids, benzophenones, and flavonoids. The second study examined seed kernels at five maturation stages using HPLC-DAD-Q-ToF-MS, revealing bioactive phenolics such as iriflophenone glucoside, maclurin C-glucoside, mangiferin, and gallotannins, with notable variations among cultivars and ripening stages [110,111].

**Figure 2:**
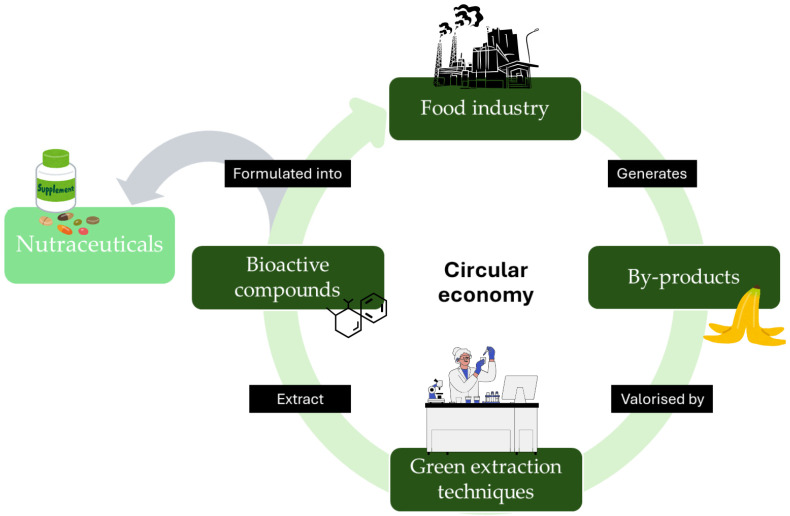
Valorisation of fruit and vegetable by-products through green extraction, recovery of bioactive compounds, and their reuse in functional foods and nutraceuticals within a circular bioeconomy.

Overall, these studies highlight the significant potential of fruit and vegetable by-products as sustainable sources of bioactive compounds for functional foods and nutraceuticals, in line with the principles of the circular economy (Figure 2). From peels, seeds, and hulls to leaves and pomace, various extraction methods have been demonstrated to recover compounds with antioxidant, prebiotic, anti-inflammatory, and antimicrobial properties. Incorporating these by-products into food formulations or dietary supplements not only valorises agricultural waste but also provides opportunities to support human health and well-being. However, safety and regulatory aspects must be considered before their use in food and supplements. Some by-products may contain anti-nutritional factors, pesticide residues, or mycotoxins, demanding careful assessment and processing to ensure safety for human consumption [112]. Technological solutions such as encapsulation and nanoemulsions are being explored to enhance the stability, bioavailability, and safety of these compounds [113]. Regulatory compliance and toxicological evaluations are essential steps in bringing by-product-derived bioactives to market [114].

**Table 2:** Summary of bioactive compounds, extraction method, intended applications, and health-related effects of selected fruit and vegetable by-products for nutraceutical and food supplement development.

By-Product Source	Main Bioactive Compounds	Extraction Method	Aim	Effects	Ref.
Peach, apricot, apple, tomato	Polyphenols: phenolic acids, flavonoids Other: proteins	UAE	Nutraceutical functional ingredients and dietary supplements	Antioxidant	[93]
Cantaloupe	Polyphenols Dietary fibre	Hydromethanolic extraction	Functional food ingredient for gluten-free bakery products	Antioxidant	[94]
*Asparagus officinalis* stem and root	Dietary fibre Insulin Low- and high-molecular-weight polyphenols	Hydromethanolic and hydroethanolic extractions	Potential prebiotic supplement or functional ingredient	Prebiotic potential for gut microbiota modulation	[95]
Turmeric	Phenolics, flavonoids, and curcumin Dietary fibre (insoluble & soluble) Minerals	Aqueous acetone extraction	Use as a functional ingredient in fortified cookies to enhance antioxidant and fibre content	Antioxidant	[96]
Oat husk and bran	Phenolic acids, mainly ferulic acid Dietary fibre	Micronization	Development of a fibre-rich antioxidant ingredient for food or dietary supplement formulations	Antioxidant	[97]
Grape pomace, bilberries, red currants	Major phenolics: Chlorogenic acid, rutin, ferulic acid, catechin	UAE	Dietary supplement in capsule form	Potential to prevent oxidative stress related chronic diseases	[98]
Apple	High total and insoluble dietary fiber Free phenolic compounds and biogenic compounds	UAE	Development of a dietary supplement	Antioxidant and anti-inflammatory Potential hypoglycaemic/antidiabetic effects	[99]
Artichoke	Hydroxycinnamates: 5-*O*-caffeoylquinic acid, 3,5-di-*O*-caffeoylquinic acid Flavonoids: apigenin-7-*O*-rutinoside, luteolin, luteolin-7-*O*-rutinoside	Aqueous extraction	Evaluation for nutraceutical and dietary supplement applications	Antioxidant Prebiotic potential	[100]
*Mauritia flexuosa*	-	Aqueous extraction	Evaluation for food supplement/nutraceutical for inflammation	Anti-inflammatory	[101]
*Citrus reticulata* Blanco peel and pomace	Catechin, neohesperidin, nomilinic acid derivatives	UAE	Functional ingredients for nutraceuticals or food applications	Antioxidant	[102]
Mango peel	Pectin	MAE	Sustainable development of prebiotics	Prebiotic	[103]
Pistachio hull	Cyanidin-3-O-galactoside, gallic acid, catechin, and eriodictyol-7-O-glucoside	Methanolic extraction	Food product applications potential	Antidiabetic, antifungal, antioxidant and anti-glycative	[104]
Guava purée	Rhamnose and xylose	Ethanolic extraction	Incorporation in yogurt-making for increased probiotics	Prebiotic	[106]
European cranberry bush and sea buckthorn berry pomace	Linoleic, linolenic, oleic, palmitic and palmitoleic acids, b-sitosterol and a-tocopherol	SFE	Potential application in functional foods and nutraceuticals	Antioxidant	[107]
Pumpkin leaves	Proteins	UAE	Potential application as food additives and dietary supplements	-	[108]
Mango peel	Mono- and di-galloyl compounds, gallotannins, phenolic acids, benzophenones, flavonoids	UAE	Potential application as nutraceuticals	-	[110]
Red pomegranate seeds	Protein and peptides	Protein extraction and enzymatic hydrolysis	Potential use in food formulations and dietary supplements	Antioxidant, antibacterial and blood pressure lowering	[109]
*Corylus avellana* hells	Lignans, flavonoids, gallic acid derivatives, diarylheptanoids and fatty acids	MAE	Nutraceutical formulations	Antidiabetic and antioxidant	[105]
Mango seed kernel	Iriflophenone glucoside, maclurin C-glucoside, maclurin digalloyl glucoside, mangiferin, 5-galloyl quinic acid, trigalloyl glucose, hexa-gallotannins, hepta-gallotannins	UAE	Nutraceutical formulations	-	[111]

MAE: microwave-assisted extraction; UAE: Ultrasound-assisted extraction; SFE: supercritical fluid extraction.

## 5. Challenges and Future Perspectives

Despite the progress achieved, the use of fruit and vegetable by-products as sources of bioactive compounds still presents multiple challenges, particularly concerning the safety, regulatory approval, and standardization of bioactive-rich extracts from food by-products. Furthermore, by-products are chemically diverse and influenced by plant variety, maturity, and processing conditions, which complicates standardization and quality control of the extracted compounds. It highlights the importance of establishing consistent specifications for bioactive-rich extracts.

Another significant challenge lies in optimising of extraction processes. While advanced green extraction methods, such as UAE, MAE, SFE, and cold plasma techniques, offer high efficiency and sustainability, their translation from laboratory to industrial scale is still in its early stages, due to issues including scalability, process reproducibility, and equipment costs. Moreover, the variability in the concentration and stability of compounds during storage and processing underscores the need for standardized extraction protocols that can preserve bioactivity and ensure reproducibility.

The bioavailability and health efficacy of many bioactive compounds recovered from by-products also remain insufficiently understood. Numerous studies have confirmed the antioxidant, anti-inflammatory, and antimicrobial properties. However, comprehensive human clinical trials validating these effects under realistic dietary conditions are still lacking. Additionally, the interactions of multiple bioactive compounds within complex matrices must be considered.

Future research must prioritize thorough characterization and standardization strategies, including the development of optimized protocols for extraction, quantification, and quality assessment. Human intervention studies are necessary to substantiate health claims and determine effective dosages. Advances in formulation strategies, such as encapsulation systems, may enhance the stability and bioavailability of these compounds, increasing their functional efficacy in dietary supplements. Finally, aligning these technological developments with the principles of the circular bioeconomy can maximize resource efficiency and also meet the growing consumer demand for natural, health-promoting, and environmentally responsible food ingredients.

## 6. Conclusions

Fruit and vegetable by-products are valuable and underexploited sources of bioactive compounds that can be efficiently recovered through green extraction technologies. Once considered waste, these residues represent a renewable resource for functional foods and nutraceuticals with antioxidant, anti-inflammatory, and prebiotic potential. Translating this potential into industrial reality requires progress in three key areas: scalability, since the most promising techniques (UAE, MAE, SFE) still face engineering and economic barriers when moving from laboratory to continuous industrial systems; safety and regulation, given the need for by-product-derived extracts to comply with food-grade standards, ensuring absence of contaminants, reproducibility, and adherence to EU and FDA guidelines for novel ingredients; and clinical validation, which requires controlled human trials to justify nutraceutical claims and establish effective doses, even with in vitro and animal studies confirming antioxidant and metabolic benefits. Future research should therefore focus on extraction optimisation, including life cycle and cost analyses, safety evaluation, and translational studies that link bioactivity to measurable health outcomes. Following these steps, by-product valorisation can evolve from an academic concept toward large-scale, safe and evidence-based applications in sustainable food production.

## Data Availability

The original contributions presented in this study are included in the article. Further inquiries can be directed to the corresponding author.
